# Structural Dynamics of Tropical Moist Forest Gaps

**DOI:** 10.1371/journal.pone.0132144

**Published:** 2015-07-13

**Authors:** Maria O. Hunter, Michael Keller, Douglas Morton, Bruce Cook, Michael Lefsky, Mark Ducey, Scott Saleska, Raimundo Cosme de Oliveira, Juliana Schietti

**Affiliations:** 1 Earth Science Research Center, University of New Hampshire, Durham, NH, United States of America; 2 International Institute of Tropical Forestry, USDA Forest Service, San Juan, Puerto Rico; 3 EMBRAPA Monitoramento por Satélite, Campinas, São Paulo, Brazil; 4 NASA Goddard Space Flight Center, Greenbelt, MD, United States of America; 5 Natural Resource Ecology Laboratory, Colorado State University, Fort Collins, CO, United States of America; 6 Department of Natural Resources, University of New Hampshire, Durham, NH, United States of America; 7 Department of Ecology and Evolutionary Biology, University of Arizona, Tucson, AZ, United States of America; 8 EMBRAPA Amazônia Oriental, Belém, Pará, Brazil; 9 Instituto Nacional de Pesquisas de Amazônia–INPA, Manaus, Amazonas, Brazil; Chinese Academy of Forestry, CHINA

## Abstract

Gap phase dynamics are the dominant mode of forest turnover in tropical forests. However, gap processes are infrequently studied at the landscape scale. Airborne lidar data offer detailed information on three-dimensional forest structure, providing a means to characterize fine-scale (1 m) processes in tropical forests over large areas. Lidar-based estimates of forest structure (top down) differ from traditional field measurements (bottom up), and necessitate clear-cut definitions unencumbered by the wisdom of a field observer. We offer a new definition of a forest gap that is driven by forest dynamics and consistent with precise ranging measurements from airborne lidar data and tall, multi-layered tropical forest structure. We used 1000 ha of multi-temporal lidar data (2008, 2012) at two sites, the Tapajos National Forest and Ducke Reserve, to study gap dynamics in the Brazilian Amazon. Here, we identified dynamic gaps as contiguous areas of significant growth, that correspond to areas > 10 m^2^, with height <10 m. Applying the dynamic definition at both sites, we found over twice as much area in gap at Tapajos National Forest (4.8 %) as compared to Ducke Reserve (2.0 %). On average, gaps were smaller at Ducke Reserve and closed slightly more rapidly, with estimated height gains of 1.2 m y^-1^ versus 1.1 m y^-1^ at Tapajos. At the Tapajos site, height growth in gap centers was greater than the average height gain in gaps (1.3 m y^-1^ versus 1.1 m y^-1^). Rates of height growth between lidar acquisitions reflect the interplay between gap edge mortality, horizontal ingrowth and gap size at the two sites. We estimated that approximately 10 % of gap area closed via horizontal ingrowth at Ducke Reserve as opposed to 6 % at Tapajos National Forest. Height loss (interpreted as repeat damage and/or mortality) and horizontal ingrowth accounted for similar proportions of gap area at Ducke Reserve (13 % and 10 %, respectively). At Tapajos, height loss had a much stronger signal (23 % versus 6 %) within gaps. Both sites demonstrate limited gap contagiousness defined by an increase in the likelihood of mortality in the immediate vicinity (~6 m) of existing gaps.

## Introduction

Gaps are a prominent feature on the tropical forest landscape and key to the dynamics and species distribution of tropical forests [1–3]. Gap phase dynamics maintain high light environments within closed forest canopies and promote natural regeneration and turnover [4,5]. While the dynamic processes of regeneration and turnover of individuals and species are the ecological rationale for the study of gaps across the landscape, gaps are often treated as static environments defined in terms of light availability or vegetation height [6,7].

The majority of tropical forest turnover occurs via small to medium gaps caused by single trees or small groups of trees [8,9]. In the Brazilian Amazon, natural catastrophic disturbances that destroy understory vegetation such as large-scale fire and wind-throw events are rare [10,11].

Oliver and Larson [5] described the structural development of forest regeneration following disturbance in four stages: stand initiation from existing seed-banks or advanced regeneration, stem exclusion via density-dependent mortality, understory re-initiation and old-growth. Although this work was focused on temperate forests, processes described are similar in the tropics [12]. Stand establishment is typically most rapid when advanced regeneration survives a disturbance event [13]. The growth of advanced regeneration is one method of gap closure, promoting understory trees to a canopy position. However, small gaps may also close via horizontal ingrowth of surrounding vegetation [14,15].

Gaps are environments where high light conditions promote high growth rates. But gaps are not only changing environments themselves, but promote change in the surrounding forest. Ray and colleagues [16] showed that gaps change the microclimate of their immediate area as well as the surrounding forest. The change in the outer canopy surface also promotes the penetration of wind into the forest understory [17]. It has been hypothesized that this increased wind, in combination with uneven growth of tree canopies, may result in increased mortality of trees surrounding gaps, or gap contagiousness [18].

For measurement purposes, gaps in the forest matrix are defined by vertical and horizontal limits [6]. The vertical limit is a maximum vegetation height, and the horizontal limit is the minimum gap size. The selection of gap characteristics in previous studies reflects the feasibility of field measurements and a synthesis of ecological concepts; in a general sense, a gap includes contiguous areas of forest canopy below the dominant canopy height that receive abundant light to promote rapid growth.

Variability in gap definitions leads to difficulty in comparing between gap studies [6]. Gaps have been defined in the field using a number of techniques, the most common of which was published by Brokaw [8]. Brokaw developed his definition in order to “understand forest composition and structure” and specifically noted “changes [to forest composition and structure] are often actuated by the creation of gaps when trees fall, occasioning the creation of new tree age classes and accelerated growth of previously suppressed individuals.” Brokaw was specifically interested in the “turnover rate” of the forest. He estimated turnover rate by dividing the total area in gaps by the total area of field surveys conducted over 5 years at Barro Colorado Island (BCI), Panama. He further stipulated a minimum gap size because gaps must be “readily distinguishable amid the complexity of forest structure” and suggested a range of 20 to 40 m^2^.

A recent review by Schliemann and Bockheim [19] of gap processes defines gaps based on tree mortality and treefall, and does not specify a minimum gap size. The Brokaw definition’s minimum size is based on whether gaps are distinguishable to the field worker from the surrounding forest canopy. Other authors found that significantly smaller gaps (down to 4–5 m^2^) could be identified within the surrounding forest [20–22]. Based on the presence of coarse woody debris, Espirito-Santo et al. [9] determined a minimum size threshold of 20 m^2^ to identify branch or tree mortality. In later work, Espirito-Santo et al. [23] referred to smaller openings in the canopy surface as sun-flecks.

Landscape scale studies of tropical forest dynamics typically rely on passive optical satellite remote sensing data that cannot resolve small and medium sized gaps [24–26]. Studies using Landat show limited success in estimating gap fraction at the pixel level (approximately 0.1 ha) but fail to capture gaps of the smallest sizes [26,27]. At higher resolution (1–4 m), passive optical images such as IKONOS are complicated by the presence of shadows [9,28]. Passive optical imagery also has to contend with the problems presented by clouds, which are prevalent in the humid tropics. At the other extreme, field plots rarely capture areas greater than 1 ha. The Center for Tropical Forest Science (CTFS) affiliated with the Smithsonian Institution operates a network of field plots, the largest of which are 50 ha [29,30]. Large plots have contributed significantly to our understanding of gap formation and recovery, yet 50 ha is still relatively small to capture landscape scale patterns of disturbance and recovery [31,32].

A promising method for analyzing both gap frequency and gap size distributions is high-resolution active remote sensing. Lidar (light detection and ranging or laser scanning) is an active remote sensing method that provides accurate height information, often from multiple laser ranging measurements per square meter. It has been used to successfully describe surface canopy roughness and forest structure at varying scales [33–35]. Recently, lidar has been used for tropical gap studies, for example, to examine size frequency distributions over large areas (400–125000 ha) [36–38].

The Brokaw gap definition has been extended to the analysis of forest structure using airborne lidar [36,38–40]. At first glance, the Brokaw [8] definition of “a ‘hole’ in the forest extending through all levels down to an average height of two meters above the ground,” should be easily applicable to lidar data and more accurate than the human observer. However, Brokaw also adds the wisdom of a field scientist to his definition, noting that “an opening can conform sufficiently to this definition despite an isolated small tree or very thin branch extending into the ‘hole.” Whereas the human observer can ignore the messiness of real forest gaps, a lidar instrument will simply register a point greater than 2 m above the ground. Root balls, fallen trunks and branches, as well as remaining standing trunks are all present after gap creation events. As we show below, the gap definition has a strong effect on estimated turnover rates. Therefore, we seek a definition of gaps applicable to lidar data that accounts for both the messiness of real forests and for their dynamics.

To develop a gap definition based on structural dynamics it is important to understand patterns of growth. Limited information is available on the rate of height growth in tropical forests, especially in naturally formed gaps. Lidar is particularly well suited to this task as it measures height accurately, and can cover large areas [41]. The high resolution of airborne lidar allows for measurements of individual tree growth and mortality as well as generalized views of the forest structure. In contrast, field studies have typically focused on a few dominant or pioneer species as opposed to properties of the gap as a whole [42–44], and field-based estimates of canopy height are imprecise, even if they are not biased [45].

Tapajos National Forest and Ducke Reserve are intensively studied field sites within the Brazilian Amazon [46–50]. While no study of small gap dynamics has been conducted at Ducke Reserve, recently a paper was published using IKONOS imagery covering 167 ha of Tapajos National Forest [9]. In 2008, airborne lidar was collected over both sites [51], with a second airborne lidar data collection approximately four years after the initial collection covering approximately 400 ha of Tapajos National Forest and 600 ha of Ducke Reserve. We leverage this data to develop a new gap definition based on forest dynamics and apply this data to analyze gap presence, formation and closure at the landscape scale. Our goals are to define the rate of gap formation, the size frequency, distribution and regrowth rates of gaps at these two contrasting forest areas by answering the following questions:

- What is an ecologically appropriate definition for gaps at the two sites?
- What is the distribution of gap area and gap size at two sites in the Brazilian Amazon?
- What is the frequency of gap creation and how long do gaps persist within a landscape?
- How does the frequency of gap creation compare to field estimates of mortality?
- Are gaps contagious?

## Methods

We analyzed multi-temporal lidar data and field measurements from two sites to evaluate gap dynamics in the Brazilian Amazon. The following sections provide information on site characteristics (2.1), lidar data collection (2.2), field measurements (2.3), and data analysis (2.4–2.8).

### Site Descriptions

#### Tapajos National Forest

The Tapajos National Forest (54°57’W 2°51’S) is a 550,000 ha reserve situated within the state of Pará, Brazil along the eastern shore of the Tapajos River. The reserve is primarily upland forest, and includes patches with canopy level palms. The dominant soils are nutrient-poor, clay, Oxisols [52]. A pronounced dry season lasts approximately five months, from July—December [48]. The most frequent form of mortality related to gap forming events is snapped trunks [9], associated with the high winds of the rainy season, consistent with other neotropical forests [3]. However, inventories of coarse woody debris production at the same location did not show a strong seasonal pattern [53].

#### Ducke Reserve

Ducke Reserve (59°57’W 2°57’S) is a 10,000 ha forest preserve managed by the National Institute for Amazon Research (INPA) bordering the city of Manaus, in the state of Amazonas, Brazil. The reserve is covered by upland *terra firme* forest with a large number of understory palms and occasional canopy level palms, especially in seasonally inundated valleys. The soils vary with the rolling topography (30–120 m.a.s.l.) with Oxisols dominant in upland areas, Ultisols on the slopes and Spodosols in the valleys [47]. These soils are acidic and low in nutrients. There is a short dry season lasting 1–3 months, generally occurring from July through September. Most trees die standing (54%) as opposed to processes of snapping and uprooting [54].

### Airborne Lidar Data

Airborne lidar data was collected in June and July of 2008 with a minimum required data density of 10 returns per m^2^ and actual mean return densities at each site near 40 returns per m^2^. A second airborne lidar data set was collected using a different lidar system in February 2012 at Ducke Reserve, and August 2012 at Tapajos National Forest (Tapajos) (Table 1) with a minimum required data density of 4 returns per m^2^. The resulting time between sampling periods was 44 months at Ducke Reserve and 48 months at Tapajos. The total area available for multi-temporal analysis was 398 ha at Tapajos and 603 ha at Ducke Reserve.

**Table 1:** Details of airborne lidar data collections.

Data Characteristics	Initial Collection		Final Collection	
	Tapajos	Ducke Res.	Tapajos	Ducke Res.
Lidar System	Leica ALS50-II	Leica ALS50-II	ALTM 3100EA	ALTM 3100EA
Flight Altitude	700–900 m	700–900 m	600 m	600 m
Divergence	15 mrad	15 mrad	25 mrad	25 mrad
Footprint Size at nadir	10 cm	10 cm	15 cm	15 cm
Pulse Frequency	118 kHz	118 kHz	50 kHz	50 kHz
Acquisition Date	06-07/2008	06-07/2008	08/2012	02/2012
Minimum return density (m^-2^)	10	10	4	4
Ground return density (m^-2^)	0.44	0.83	0.49	0.19

Canopy height models (CHMs) and Digital Terrain Models (DTMs) were produced from LAS files provided by our commercial data providers (Table 1) using the processing methods developed for NASA Goddard’s Lidar, Hyperspectral, and Thermal Airborne Imager (G-LiHT) [55,56]. This methodology separated vegetation and ground returns to develop a gridded representation of the ground surface (DTM) and height estimates of lidar returns from canopy and understory vegetation (CHM). Data for the Tapajos site were processed separately for each data collection. Topographic effects at the Tapajos site are minimal, and the difference between DTM layers for 2008 and 2012 lidar collections was trivial. At Ducke Reserve, we produced a unified DTM based on both years’ data [41,57]. This processing approach provided the most robust estimate of ground topography from which to generate CHM data layers (1 m horizontal resolution) for each year.

### Field Surveys

Forest inventory data from 2009 and 2011 provided field estimates of mortality and canopy turnover. Diameter-dependent line sampling was conducted along six 500 m transects at Tapajos National Forest and five transects at Ducke Reserve. Initial field surveys were conducted in June 2009 (Tapajos) and October 2009 (Ducke Reserve) and over 1000 trees were sampled at each location [45]. Permits for field work were obtained from the Instituto Chico Mendes (ICMBio) for work conducted at Tapajos National Forest and Instituto Nacional de Pesquisas da Amazônia (INPA) for work at Ducke Reserve. Live trees as well as standing dead stems greater than 5 cm diameter were included in each survey. For all living stems, the crown radius was measured in each of four cardinal directions. A circular crown area was estimated for each stem based on the mean crown radius. A weighted mean crown radius was calculated for each site, taking into account the diameter-dependent sampling. Transects were resampled in July 2011 at Tapajos National Forest and October 2011 at Ducke Reserve. Percent mortality was estimated from trees that died between samples and corrected to an annual value. The fraction of fallen versus standing dead trees was estimated based on the height measurements for dead stems. A non-parametric bootstrap analysis [58] was used to calculate 95% confidence intervals of annual mortality.

### Height Structure

Distributions of lidar canopy heights were compared for all sites and years. The height structure of the canopy was investigated using a random sub-sample of 1000 heights from each CHM, and comparisons between years were repeated 1000 times. Comparisons were also made between sites using the same technique. Sites and years were tested for significantly different height structures with a two-sided Kolmogorov-Smirnov test (R version 3.0.1). P-values reported are the average of 1000 tests. Spatial autocorrelation within the canopy height models was evaluated using a variogram (R version 3.0.1), where the range was defined as the distance at which 95% of the asymptote of semivariance (sill) was attained.

### Gap Definitions

We defined a gap using a dynamic measure of height change and a static minimum horizontal extent. We refer to this *dynamic gap* as a region characterized by significant vertical growth that accounts for the transient nature of gaps and the high light environment that exists at low canopy heights. The horizontal extent was defined as the mean crown area of all trees > 5 cm dbh at each site. To determine regions of significant vertical growth, vertical height changes were evaluated as a function of initial canopy height. To examine the height change at both sites we took a randomly distributed subset of 60 pixels (1 m^2^) from each initial integer height. At the most infrequent heights this is approximately a 1% sample and resulted in an average minimum distance of 22 m between sample points at Tapajos and 27 m at Ducke Reserve. Though spatial autocorrelation was not completely avoided using this procedure, the sampling approach reduces its potential influence. Grid sampling of pixels at a scale determined by spatial autocorrelation (26 m at Tapajos and 17 m at Ducke) was not applied as it reduced the sample size for low heights (rare at both sites) to zero. Tukey’s HSD test was used to compare height changes between initial height subsets. The entire procedure applied to determine the dynamic height cutoff was run 100 times for each site to assess variability. We compared this definition to the Brokaw [8] definition, because the latter has been most commonly used.

### Distribution of Gap Areas

Gap areas were identified in lidar CHM data as clusters of adjacent 1 m pixels that satisfied the Brokaw or dynamic gap definitions. Gaps classified in each temporal acquisition were then used to calculate the size frequency distribution of gaps following a modified application of Clauset et al [59]. This technique fits gap size distributions to a power-law using a maximum likelihood estimator [24,60,61] and a fixed minimum gap size. Gaps were also tested for the degree of spatial autocorrelation using Moran’s I (Arc 10.1), an index that ranges from -1 (indicating perfect dispersion) to 1 (perfect correlation), with zero representing near perfect randomness.

### Gap Creation and Lifetimes

Gap persistence times were calculated using two methods and two gap definitions, the Brokaw [8] gap definition and the dynamic gap definition. The total area of new gaps was used to estimate the gap recurrence interval (*t*_*r1*_), calculated using Eq 1:

$$t_{r1}=\;t_{s}\frac{A_{undisturbed}}{A_{gap}}$$ (1)

Eq 1 estimates the gap recurrence interval based on the time interval between lidar acquisitions (t_s_) and the non-gap area (A_undisturbed_) that became gap area (A_gap_) over the sampling period. This approach excluded gaps that could have both formed and closed during the sampling interval. We compared gap recurrence intervals calculated from multi-temporal lidar data (Eq 1) with estimates based on a single date of lidar coverage (t_r2_, Eq 2):

$$t_{r2}=\;t_{p}\frac{A_{total}}{A_{gap}}$$ (2)

Using a single lidar collection, gap recurrence intervals can be estimated as the ratio of the total area of the site (A_total_) to the gap area (A_gap_), adjusted for gap persistence (t_p_), where t_p_ is defined as average rate of gap closure based on measurements of average height changes (gains and losses) of trees in gaps. Despite the potential for increased bias in Eq 2, this second calculation of recurrence interval is important for comparison with existing literature. Additionally, both equations assume that the gap formation is stochastic and spatially independent. Therefore, estimates of gap recurrence interval are subject to tests of gap contagion discussed in Section 2.8.

The height change of each gap pixel was used to separate vertical height growth of trees in gaps and horizontal encroachment of neighboring crowns between lidar collections. Height growth in gaps was estimated using gap centers, areas >5 m from the gap edge at Ducke Reserve and >10 m from gap edges at the Tapajos site. These distances were more than twice the amount of lateral growth measured in temperate forests over a comparable time period [62]. The mean and standard deviation of height change were calculated for gap center pixels, and the maximum vertical growth was conservatively estimated as mean height change plus three standard deviations. This estimate of maximum vertical growth in gaps is consistent with the assumption that horizontal ingrowth of neighboring crowns is unlikely at large distances from gap edges. Height changes in gaps that exceeded this expected maximum vertical growth were attributed to horizontal ingrowth. Areas of height loss were excluded from this analysis, as these were considered evidence of repeated disturbance in gap areas. The proportion of area within each growth category (horizontal ingrowth, vertical growth and height loss) was compared with distance from gap edge and gap size.

### Gap Contagiousness

We defined gap contagiousness as the increased risk of disturbance around existing gaps [18]. Jansen et al. [18] proposed three hypotheses specific to contagiousness: (H1) canopy disturbance risk decreases with increasing distance from gaps, (H2) canopy disturbance risk is elevated in the edge zone of existing gaps, and (H3) gap bordering trees have increased risk of mortality.

To assess whether canopy disturbance risk decreases with increasing distance from gaps (H1) or is elevated in the edge zone of existing gaps (H2), we first calculated the minimum distance between all non-gap pixels and the nearest gap in the initial acquisition. We then separated the distances to pixels classified as gaps in the 2012 lidar data collections. The frequency of each distance was calculated, and the distribution of distances to new gap pixels was subtracted from the distribution of distances across the acquisition. A consistent decrease in the difference between these distributions would support the first hypothesis. A Kolmogorov-Smirnov test was applied to test the second hypothesis.

Jansen, et al. [18] originally applied these three hypotheses to field data on 5660 trees greater than 10 cm diameter collected over a 12 ha area. Because we do not have field data for the full extent of the lidar data collection, we applied a modified test for the third hypothesis. As opposed to testing the effect of gaps on all stems, we tested the effect of gaps on emergent stems (greater than 40 m tall) so that canopies and partial canopies that were present in 2008 but not in the second data collection were tallied. Canopies for which any point was within 10 m of gaps in the 2008 data set were considered to be near gaps.

## Results

### Variability of Forest Structure

**Fig 1:**
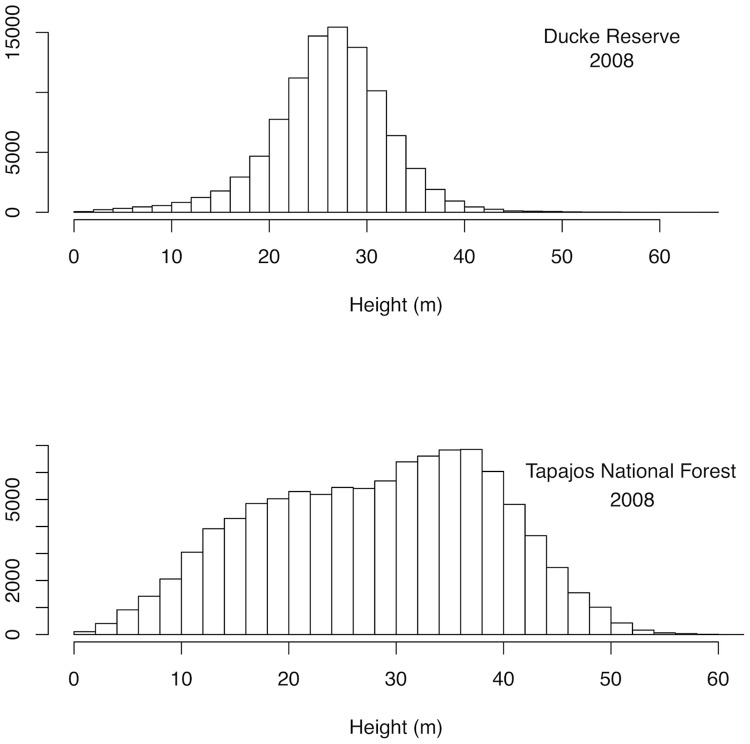
Distribution of canopy heights in 2008 airborne lidar acquisitions. Shown for (a) Ducke Reserve and (b) Tapajos National Forest.

Lidar estimates of canopy heights at Ducke Reserve had a near Gaussian distribution with a mean canopy height of 26 m (Fig 1). In contrast, Tapajos National Forest had a skewed distribution of heights that was significantly different from the distribution of heights at Ducke Reserve (KS-test p-value < 2.5e-12). The dominant canopy at Tapajos National Forest was between 35 m and 40 m, but there was a sub-dominant layer with near equal frequency from 15–30 m (mean of 28 m). Both the mean canopy height and the 99th percentile canopy height were higher at Tapajos (99th percentile height of 54 m as opposed to 49 m). Canopy height distributions were not significantly different between time periods (KS-test p = 0.05 at Ducke and 0.4 at Tapajos). The consistent difference in canopy structure may reflect the difference in mortality rates between the two sites. Annual mortality at Tapajos was estimated from field surveys as 2.1% with a 95% confidence interval of 1.4%- 2.8%. At Ducke Reserve, annual mortality was 1.4%, with a 95% confidence interval of 0.8%- 2.1%. Both sites showed spatial autocorrelation among canopy heights. At Tapajos National Forest the range of spatial autocorrelation was 26.4 m and at Ducke Reserve the range was 17.3 m. These ranges are consistent with the diameter of emergent crowns at each site.

### Dynamic Gap Definition and Minimum Gap Size

**Fig 2:**
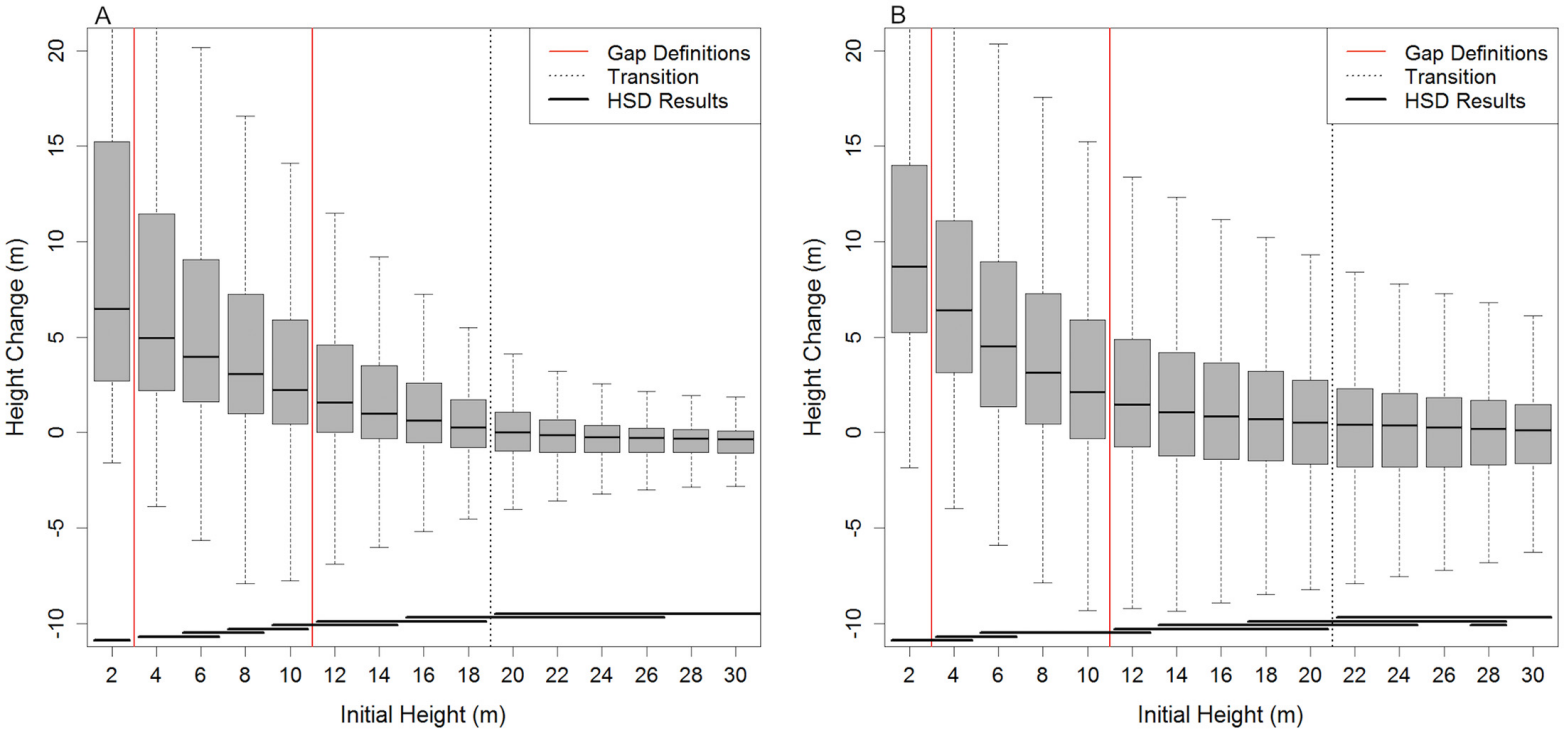
Mean and confidence interval of height change between initial and final lidar data acquisitions. Data collected at (a) Ducke Reserve and (b) Tapajos National Forest overlaid with the Brokaw (1982) and dynamic gap definitions’ height cutoffs, and the transition height where height change is not significantly different from zero based on Tukey’s HSD (horizontal segments at the base of the figure). Each horizontal line displaying Tukey’s HSD results spans initial height bins for which there is no significant difference (p-value > 0.05).

Similar to traditional gap definitions, we used a height cutoff to determine gap areas. This height cutoff was derived based on observed height changes. Height change between the initial and final lidar data collections varied with the initial vegetation height (Fig 2). The mean height change decreased exponentially with increasing initial height and became consistently less than zero above 20 m initial height at Tapajos National Forest and 18 m at Ducke Reserve (defined as the transition height). The distribution of height change of all lower initial height bins were compared with the transition height (Fig 2). We defined the height cutoff for our dynamic gap definition as the tallest initial height that showed significantly greater change in height than the transition class (Tukey’s HSD p-value < 0.05). In other words, these were the areas showing a statistically significant signal of height increase. This cutoff height was 10 m at both Tapajos National Forest and Ducke Reserve. *Gaps were defined as contiguous areas of the CHM with height less than 10 m at both sites*.

The minimum gap size (m^2^) was defined as an approximation of the mean canopy area for trees greater than 5 cm diameter at breast height (DBH = 1.3 m). The calculation of mean canopy radius took into account the basal-area weighting of the initial sample. The mean radius length was 2.07 m for trees at Tapajos National Forest and Ducke Reserve, corresponding to an estimated crown area of 13.4 m^2^. This was approximated as 10 m^2^ for ease of application and applied to both the Brokaw and dynamic gap definitions. *Thus our dynamic gap definition was defined as an area > 10 m* ^*2*^ *where the lidar measured canopy height is always < 10 m*.

Gap area and recurrence interval estimates were robust to the minimum gap size estimate. Gap area based on the 2008 Tapajos lidar data increased linearly for gap sizes from 4 m^2^ (4.6%) to 20 m^2^ (3.9%), increasing the gap recurrence interval by approximately 2 years per m^2^ increase in minimum size.

### Gap Area and Spatial Distribution

Tapajos showed a larger proportion of forest area in gaps at both time periods using both definitions. Estimated gap area at Tapajos varied between 4.1–5.5% (Table 2). Using the dynamic gap definition, 1.7 to 2.2% of the Ducke Reserve was in gaps—less than half the gap area at Tapajos National Forest. Both sites showed a larger percentage gap during the second sampling (Table 2). Gaps at Tapajos showed no statistically significant spatial autocorrelation (I = 0.004; p-value = 0.5). At Ducke Reserve, gaps showed a weak spatial autocorrelation (I = 0.05; p-value = 0.01).

**Table 2:** Frequency of gap formation presented for both sites and gap definitions.

Gap Definition	Site	Sample Area (ha)	Initial Gap Area (%)	Final Gap Area (%)	Percent New Gap (%)	KS-test p-value
Dynamic Gap	Ducke Reserve	602	1.20	1.52	64.2	0.39
	Tapajos	398	4.37	5.49	23.2	0.33
Brokaw (1982) Gap	Ducke Reserve	602	0.01	0.04	98.9	0.78
	Tapajos	398	0.03	0.11	98.8	0.95

The area covered by lidar at both time periods is reported with the proportion of initial gap area, proportion of final gap area, and the proportion of the amount of final gap area newly formed between samples for both the dynamic gap definition (10 m height cutoff) and the Brokaw (1982) gap definition (2 m height cutoff) for a minimum gap area of 10 m^2^. A Kolmogorov-Smirnov test was used to compare the distributions of gap sizes between years for each site by definition.

The distribution of gap sizes also differed significantly between the two sites (KS test p-value < 0.01). Dynamic gaps at Ducke Reserve were smaller, on average (35 m^2^) than at Tapajos (68 m^2^), with maximum gap sizes of 0.05 ha at Ducke Reserve and 0.9 ha at Tapajos. The exponent of the gap size power-law distributions averaged 2.16 at Ducke Reserve with 95% confidence intervals from 2.12–2.20. At Tapajos National Forest the exponent was 1.88 using the dynamic gap definition for both years (95% confidence interval of 1.84–1.90).

Gaps that satisfied the Brokaw gap definition (<2 m height) and 10 m^2^ minimum area were rare, yet the analysis of Brokaw gaps preserved the relative differences between sites (Table 2). Power law exponents of the gap size distributions were significantly greater for Brokaw gaps (3.26 and 2.91 at Ducke Reserve for 2008 and 2011 respectively and approximately 2.86 at Tapajos National Forest for both years). Kolmogorov-Smirnov tests showed that gap size distributions did not differ between years (Table 2).

### Gap Creation and Lifetimes

Using the dynamic gap definition, new gaps that formed between lidar collections at Ducke Reserve accounted for 65% of all gap area in 2012 (t_s_ = 3.67 years). At Tapajos National Forest 23% of gap area was formed between the lidar collections, a period of 4 years. Despite differences in the proportion of new gap area, the recurrence intervals calculated using Eq 1 were similar: 377 years at Ducke Reserve and 316 years at Tapajos National Forest.

The persistence time of gaps was calculated based on the height changes between lidar collections (Table 3). Height losses within gaps totaled 13.4% of gap area at Ducke Reserve and 22.6% at Tapajos National Forest. Losses in tree heights within gaps may represent repeated disturbances, delayed mortality, or decomposition following tree-fall events. Taking repeat disturbance into account resulted in estimated annual growth rates of 1.23 m y^-1^ at Ducke Reserve and 1.10 m y^-1^ at Tapajos National Forest. Estimated recurrence times calculated from a single lidar acquisition (Eq 2) were more variable than direct estimates of recurrence times from multi-temporal lidar. At Ducke Reserve, the recurrence times for dynamic gaps were 675 years and 532 years for the initial and final acquisitions respectively. At Tapajos, recurrence times were 208 years and 165 years.

**Table 3:** Estimated gap recurrence frequencies based on gap persistence from multi-temporal lidar data.

Gap Definition	Site	Persistence Time y (t_p_)	Recurrence Time y (t_r_)		
			New Gaps between 2008–2012 lidar ****(Eq 1)****	2008 Lidar (Eq 2)	2012 Lidar ****(Eq 2)****
Dynamic Gap	Ducke Reserve	8.1	371	675	532
	Tapajos	9.1	301	208	165
Brokaw (1982)	Ducke Reserve	0.9	9009	7416	2122
	Tapajos	0.8	3725	2367	732

Inter-sample period growth takes into account both height gain and height loss in gap areas. Three recurrence frequencies are presented: (1) Taking into account only areas that were not in gap in the 2008 lidar scene (Eq 1), (2) Using the entirety of the 2008 lidar scene (Eq 2), (3) Using the entirety of the 2012 lidar scene (Eq 2)

Using the Brokaw [8] definition, nearly 100% of the gap area in 2012 formed between lidar acquisitions. Recurrence intervals of Brokaw gaps were more than ten times longer than estimates at both sites based on the dynamic gap definition. At Ducke Reserve the recurrence interval was 9,009 years, longer than the estimated 3,725 years at Tapajos National Forest. Growth was estimated as 2.36 m y^-1^ at Ducke Reserve and 2.52 m y^-1^ at Tapajos for initial heights less than 2 m. This rapid height change resulted in persistence times of less than a year at both sites. Recurrence intervals calculated for individual acquisitions were extremely variable.

Horizontal ingrowth accounted for only 6–10% of gap closure between the lidar acquisitions. The mean positive height change per year at gap centers (assumed unaffected by horizontal ingrowth) was 1.2 m y^-1^ (sd = 0.9) at Ducke Reserve and 1.8 m y^-1^ (sd = 0.7) at Tapajos National Forest. Maximum vertical growth of approximately 4 m per year at each site captured all gap center height changes. We estimate that 9.8% of gap area at Ducke Reserve closed through horizontal ingrowth and 6.1% of gap area at Tapajos National Forest.

Height loss (repeat disturbance) accounted for a larger proportion of gap area than horizontal ingrowth at both sites, accounting for 13.4% of gap area at Ducke Reserve and 22.6% of gap area at Tapajos National Forest. However, this percentage was not evenly distributed within gaps. Analyzing the region within 5 m of gap edges, an increase in repeat disturbance was observed at Tapajos (26.9% of area) and a decrease at Ducke Reserve (10.6%). When height loss was included, the average height change at gap centers was similar to the overall height change of gaps (1.1 m y^-1^ at Ducke Reserve and 1.3 m y^-1^ at Tapajos).

### Gap Contagiousness

**Fig 3:**
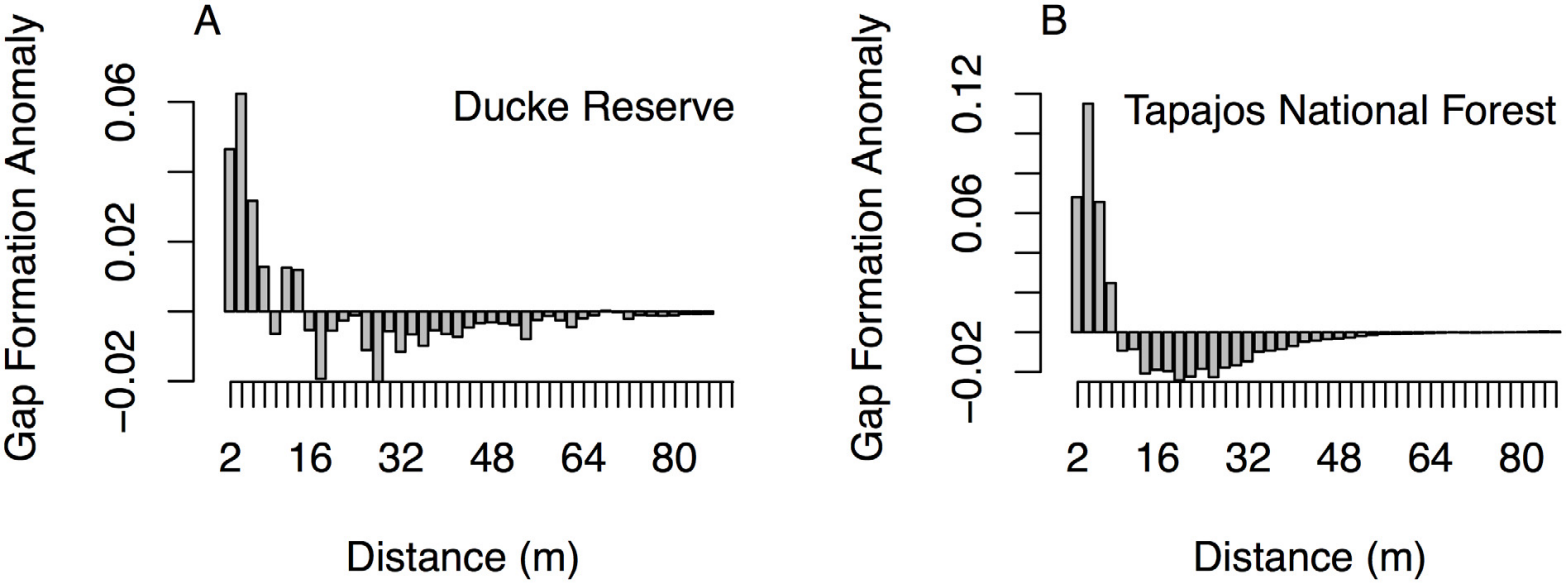
Gap formation anomaly with distance from existing gaps. For each site, the average probability of gap formation between 2008 and 2012 was calculated and subtracted from the probability of gap formation based on distance from existing (2008) gaps. The resulting difference (Gap Formation Anomaly) was plotted against distance for (a) Ducke Reserve and (b) Tapajos National Forest.

We found limited evidence for gap contagiousness. The probability of canopy disturbance did not consistently decrease with increasing distance from dynamic gaps at either Ducke Reserve or Tapajos National Forest. At Ducke Reserve there was an increased probability of gap formation (compared to site mean) at distances of less than 10 m from existing gaps (Fig 3a). At Tapajos National Forest, this increased probability extended to 8 m from existing gaps (Fig 3b). The increased probability of gap formation near to existing gaps was statistically significant (KS test p < 0.01). The strongest effect was within 6 m of existing gaps, which accounts for 7.7% of area surveyed at Ducke Reserve and 18.6% of area at Tapajos National Forest.

Gap contagiousness was further supported by our analysis of emergent tree mortality. At Tapajos National Forest we found 177 full crown and a further 74 partial crown mortality events. Of these events, 49% were within 10 m of existing dynamic gaps, compared to 34% of the data that were within this same distance. At Ducke Reserve, we found 114 full crown mortality events, and 34 partial crown events. Of these mortality events 20.3% were within 10 m of dynamic gaps, and 16% of the area sampled. Observations showed higher than expected mortality near gaps at both sites with a 15% difference at Tapajos National Forest and a 4% difference at Ducke Reserve (observed—expected). At both sites, lidar based estimates of emergent mortality are not significantly different from overall site mortality based on field surveys (Table 4).

**Table 4:** Estimates of annual mortality based on field and lidar samples.

	Ducke Reserve				Tapajos National Forest			
	N_m_	N	% Ann. Mortality	% Fallen	N_m_	N	% Ann. Mortality	% Fallen
All Field	23	899	1.4	53.0	49	1,137	2.1	59.1
Field Emergents	0	16	—	—	3	61	2.9	28.9
Lidar Emergents	114	1,583	1.9	—	177	2,082	2.1	—

Field based estimates were calculated for all trees as well as emergent trees (>40 m). Lidar-based estimates of annual mortality are for emergent trees only (>40 m). Columns indicate the number of dead trees (N_m_), total sample size (N), and the proportion of annual mortality between field or lidar sampling intervals. Fallen dead trees had heights <10 m in the 2011 survey.

## Discussion

### Variability in Forest Structure

Given the variability in the dates and periods between lidar scans, it is possible that leafing patterns may affect the CHMs used for analysis. A study conducted by Malhado et al. [63] in intact forest at Tapajos National Forest showed variability in LAI of 8% between the months with the highest LAI (December) and lowest LAI (April). However, work by Doughty and Goulden [64] at a logged site within Tapajos National Forest found seasonal variability in LAI in the range or 20%. Neither study addressed inter-annual variability that may be important in the multi-year data set examined. Studies monitoring litterfall near Ducke Reserve show two average peaks of litterfall, one during the dry season (Aug-Oct) and a secondary one during the rainy season, in March [65]. This seasonal pattern however, is irregular among years depending on winds and intense rains occurrence [66]. At Tapajos, lidar data was collected at the same time of year in 2008 and 2012. At Ducke Reserve, the lidar collections were in June and February respectively, both of which precede seasons of peak litterfall. We cannot rule out the possibility that LAI variations significantly affected our results but there is no indication of this effect in our data. For example, the distribution of heights sampled did not change between sampling periods at either site (Komorgorov-Smirnov test, p = 0.4 at Tapajos and p = 0.05 at Ducke).

### Gap Definition

We sought a functional gap definition based on site-specific forest dynamics in the form of measurable canopy growth. It is not expected that a single set of horizontal or vertical limits will be appropriate for all sites because of different dynamics. The two sites studied show large variability in canopy structure, but analysis of canopy dynamics resulted in one consistent gap definition for both sites. This may be due in part to their similarities as moist tropical forests. Both the vertical and horizontal limits derived are within the ranges applied in previous studies. The height limit is close to those applied in the field by Young et al. [67] and to remote sensing data by Gaulton et al. [68]. We emphasize that our quantitative dynamic gap definition can be calculated for any forest site where appropriate multitemporal data is available.

In comparison to the Brokaw definition, we find that application of our dynamic gap definition to lidar leads to much greater proportion of gap area and therefore much shorter gap recurrence intervals (Table 2). A previous remote sensing study on gap frequency in Peru does not present canopy openness or gap recurrence intervals, but tests the gap size distribution of the Brokaw and other gap definitions [40]. They showed no significant difference between the Brokaw gap definition and other height cutoffs when examining the gap size distribution in Peru [40]. In contrast, we observe significant differences in this variable. These differences may be due to the shape of gaps, annual variability, or the infrequency of newly formed gaps penetrating to within 2 m of the ground at our sites. Another important difference in our methods from those of Boyd et al. [40] is the horizontal size limit applied. While Boyd applied a 2 m^2^ minimum gap size, we applied a 10 m^2^ minimum gap size. We consider 2 m^2^ unrealistic from an ecological perspective. Brokaw [8] recommended a range from 20 to 40 m^2^ based on his field observations. As compared to Boyd, our larger minimum area reduced the total area of Brokaw gaps significantly (from 3824 m^2^ with 1 m^2^ minimum gap area to 709 m^2^ at Ducke Reserve in 2008). However, even when no minimum gap size is applied differences in gap area between the Brokaw and dynamic gap definitions remained significant (0.01% Brokaw gap versus 1.9% dynamic gap at Ducke Reserve).

The presence of standing dead within new gap areas in addition to root balls and woody debris are expected to strongly influence the ability to remotely sense canopy gaps with lidar using the Brokaw definition. As shown by Hubbell and Foster [30], the death of canopy trees often does not indicate the death of all understory vegetation. They reported that small stems frequently remained in gaps up to 4–5 m in height. Lieberman et al. [69] reported broken and damaged stems within a tree fall gap that were well above 2 m. Of the trees that were broken and died, they reported that all were less than 10 m tall (maximum 7 m). Of those that were broken and survived two were above the 10 m threshold applied here.

The minimum area of gaps delineated had a comparatively small effect on the gap characteristics presented here. Starting from a minimum area of 4 m^2^ we found that a 1 m^2^ increase in the minimum gap size results in a 0.1% decrease in total gap area and an approximate 2 year increase in recurrence interval. In contrast, the fractional area changes significantly when the height limit of the gap definition changes. Lobo et al. [37] also showed an exponential increase in the percent area in gap when the height limit is increased.

### Gap Area

We measured about twice as much proportional area in gap at the Tapajos National Forest as compared to Ducke Reserve. The Tapajos National Forest distribution of gap sizes was weighted more strongly toward large gaps. Compared with other tropical sites including Ducke Reserve, Tapajos National Forest has a more varied canopy structure, but the distribution of gap sizes is similar to previously published values [24,36,37,39,40]. Unfortunately, the proportion of area in gap is not always reported in gap studies. Of sites with similar measurements, a study conducted at Tambopata [40] that applies a minimum gap size of 2 m^2^ reported proportion of area in gap and found that approximately 1.1% of the area studied was considered gap when applying the Brokaw gap definition, higher than at either of the sites considered here. In contrast, a study conducted in Panama using a 5 m^2^ minimum gap size found 0.41% of area in gap when applying the Brokaw gap definition, and 6.04% of area in gap when applying a 10 m height cutoff [37].

A study by Poorter et al. in the Ivory Coast showed clumping of gaps across the landscape [70], whereas neither site studied here showed strong spatial correlation. The African study showed correlation between gap occurrence and slope position, with upper and middle-slope positions more likely to have gaps than on crests or lower-slope areas. The effect of slope position may explain the slight difference in clumping patterns between Ducke Reserve and Tapajos National Forest. No clumping was found at Tapajos, whereas slight clumping was found at Ducke Reserve where there is significantly more topographic relief.

### Gap Creation and Forest Turnover

Within Central America and Africa, gap persistence has been estimated from field studies as between 2 and 5 years. Hubbell and Foster [30] estimate gap persistence at Barro Colorado Island, Panama at 3.6 years. Within the Brazilian Amazon, gap persistence of a few months was found [71]. This is consistent with the result presented here of maximum height change within gap centers of approximately 4 meters per year. Any gap closure rate will be strongly influenced by the distribution of gap sizes measured. Whether the estimate within the Brazilian Amazon was biased due to the artificial creation of gaps is unclear, though an important component in the rapid closure of gaps was lateral ingrowth from surrounding trees. Variability in persistence times of gaps in the Brazilian Amazon significantly alters estimated gap recurrence intervals.

It is not expected that rates of forest turnover will be the same when estimated from individual tree mortality and from gap events because not all tree mortality will create gaps [6,69,72–76]. However, large differences are equally unrealistic. Inventories of trees within the Brazilian Amazon show annual mortality rates of 1–2%, with one-third of mortality events resulting in tree fall and potential gap creation [9]. Given an equal distribution of mortality mode across tree sizes, this would result in approximately 0.5% annual gap formation. Given persistence times of a few months, the measurable gap fraction is expected to be 0.1–0.3%. However, the expected gap fraction is 1.8% when using the persistence time of 3.6 years suggested by Hubbell and Foster [30]. By these estimates, the gap fractions of Brokaw gaps found at Ducke Reserve (0.01–0.04) and Tapajos National Forest (0.03–0.1) are far below expected values.

The unrealistically low gap fraction based on the Brokaw definition supports our contention that the debris present at the time of gap creation may significantly affect our ability to remotely sense gaps. The detritus from the gap creation events has historically not been taken into account, possibly because it can be easily ignored by the field worker. Root balls, fallen trunks and branches as well as remaining standing trunks are all present after gap creation events. While the size and presence of coarse woody debris has been studied, little to no information is available on the placement of this woody debris above the forest floor.

In contrast, the recurrence interval estimated based on the dynamic gap definition is somewhat longer than expected based on literature from Central America and Africa. Recurrence intervals in other tropical ecosystems are 60–240 years, with Central American sites ranging from 100–150 years (reviewed in Jans et al. [77]). These differences are likely due to site differences between the Brazilian Amazon and other regions. Using radiocarbon dating in combination with size distributions of stems has shown mean forest ages of 240 near Ducke Reserve, and 140 years at Tapajos [48]. These ages are less than the estimated recurrence intervals based on the dynamic gap definition (370 years at Ducke Reserve and 300 years at Tapajos) but we caution that much tree mortality does not cause gap formation so gap area based recurrence intervals are likely to be longer than those based on mortality statistics.

The differences between recurrence intervals as estimated from change between lidar data collections versus those based on single acquisitions highlights uncertainties due to the variability in persistence times. The increasing availability of repeat collections of lidar data in tropical as well as temperate ecosystems will improve our understanding of growth at the stand and landscape scales for gap and non-gap environments.

The increased gap creation, faster dynamics and larger gap sizes of Tapajos National Forest should also be associated with faster regrowth within gaps. However, we observed slower average vertical growth in gaps at Tapajos National Forest compared to Ducke Reserve. Larger gap sizes at Tapajos National Forest are expected to result in higher light availability at Tapajos National Forest, resulting in faster growth [51]. We attribute the lower observed average height change to the strong influence of repeat disturbance at gap edges, especially for gaps over 50 m^2^. While Tapajos and Ducke Reserve show similar levels of repeat disturbance for gaps less than 50 m^2^ (approximately 15%), gaps greater than 50 m^2^ have higher repeat disturbance rates at Tapajos (28% within 5 m of gap edges) and lower rates at Ducke Reserve (10% within 5 m of gap edges). Additionally, gaps are smaller at Ducke Reserve, and horizontal ingrowth has a stronger effect with a conservative estimate of 10% of gap area closing via horizontal ingrowth versus 6% at Tapajos National Forest. Furthermore, when only positive change data from gap centers were analyzed, maximum vertical growth rates are consistent with published values from other tropical forests [43] and our result of larger average height change at Tapajos National Forest is consistent with the expectations of faster dynamics at this site [51].

### Gap Contagiousness

It is debatable whether gaps influence the creation of other gaps (also known as gap contagiousness). Young and Hubbell [67] surveyed large trees within Barro Colorado Island and hypothesized that gap contagiousness would occur over time due to observed canopy asymmetry. Jansen, et al. [18] tested four hypotheses related to canopy disturbance risk and the magnitude of canopy disturbances with relation to proximity to gaps and initial gap size within a 12 ha area of tropical forest in French Guiana. While new gaps formed more frequently close to existing gaps, the authors showed that this was due to the respective area at each distance from gaps within the landscape, and was not evidence for gap contagiousness. The authors concluded that in the forests of French Guiana surveyed newly formed gaps were consistent with previous theories of tropical rain forests as “patches with predictable regeneration cycles”.

Although gap contagiousness has not been conclusively demonstrated, edge effects are well known phenomena in fragmented landscapes. Laurance et al. [17] showed increased mortality within 100 m of the forest edge, and suggested that this may be due to increased wind turbulence and changes in the local microclimate. While natural gaps within the forest matrix may not experience increased wind turbulence, changes in the local microclimate do occur [16].

Our results suggest that gap contagiousness does occur surrounding natural forest gaps but has an extremely small effective range. This was shown for all gaps and specifically in terms of the mortality of large trees. Where gap contagiousness occurs the assumption of stochasticity of gap formation necessary to the calculation of gap recurrence interval is not correct. The small effective range suggests that the gap definition used will have a strong effect on evidence for or against contagiousness. Jansen uses an expanded gap definition that is based on the Brokaw definition of a gap as a region where vegetation does not exceed 2 m height. The expanded gap definition, based on Runkle [78] requires a central area of greater than or equal to 4 m^2^ of less than 2 m height, but the gap edge is defined by the trunk locations of surrounding trees of at least 20 m in height. Young and Hubbell [67] use a different gap definition where gap areas were defined as areas greater than 25 m^2^ with canopy height less than 10 m. Jansen and colleagues’ gap definition will therefore include larger areas, but classify fewer gaps. This is apparent in their results that show mortality rates approximately twice as high within gaps as compared to the surrounding forest [18].

In summary, we found that forest canopy structure is significantly different between the two sites studied in the Brazilian Amazon. Additionally, the growth rates within gaps were highly dependent on the initial height of vegetation examined. For vegetation less than 10 m in height, we observed average height changes of approximately 4.5 m at both sites. This equates to 1.2 m y^-1^ at Ducke Reserve and 1.1 m y^-1^ at Tapajos National Forest. With regards to gaps, the gap size frequency was significantly different between the sites, as well as between gap definitions. The gap size frequency did not change between sample years, although the proportional area in gaps varied between years, suggesting that rates of canopy turnover are not constant through time. Both sites showed evidence of gap contagiousness, although the range of influence was extremely limited which may account for conflicting results in the literature.
